# Composition of two‐week change in body weight under unrestricted free‐living conditions

**DOI:** 10.14814/phy2.13336

**Published:** 2017-07-04

**Authors:** Surabhi Bhutani, Eva Kahn, Esra Tasali, Dale A. Schoeller

**Affiliations:** ^1^ Department of Nutritional Sciences University of Wisconsin Madison Wisconsin; ^2^ Department of Neurology Northwestern University Feinberg School of Medicine Chicago Illinois; ^3^ Department of Medicine The University of Chicago Chicago Illinois

**Keywords:** Body composition, dual energy X‐ray absorptiometry, energy density, fat‐free mass, fat mass, total body water

## Abstract

The composition of weight change has a large impact on energy balance calculations. Composition of long‐term weight change interventions is well‐documented, but information on short‐term weight change under unrestricted free‐living conditions is limited. The composition and energy density of the changes in body weight during 2‐week free‐living conditions were analyzed in adults from two cohorts: cohort 1 (*n* = 24) included participants from the reproducibility subset of the Observing Protein and Energy Nutrition study; cohort 2 (*n* = 22) included participants who were studied under free‐living conditions in an ongoing study in the Chicago area. Change in body weight, total body water (TBW) by stable isotope dilution (cohort 1), and fat mass (FM) and fat‐free mass (FFM) by serial DXA (cohort 2) were measured. To determine the fractional composition of the change in body weight we analyzed the linear associations between changes in body weight and changes in body composition. In the combined dataset, the average change in body weight (0.26 ± 1.2 kg) was consistent with being in energy balance. Average change in body weight was associated with the change in TBW (*P* < 0.0001) in cohort 1 and the change in FFM (*P* = 0.0002) in cohort 2. A unit change in body weight was composed of 84% change in FFM in the combined dataset indicating that 2‐week fluctuation in body weight is largely composed of FFM. The energy density of 1–3 kg short‐term changes in body weight averaged 2380 kcal/kg.

## Introduction

Energy balance, as summarized by the energy balance equation, is described by three terms; energy intake, energy expenditure, and change in body energy stores (Hall et al. 2012). Because energy cannot be created nor destroyed, this energy balance equation is widely applied to assess the third component when the other two components of energy balance are measured. Under highly controlled conditions, changes in body energy stores and hence energy balance can be calculated by subtracting energy expenditure from energy intake (Schoeller and van Santen 1982; Kempen et al. 1995; Racette et al. 1995). Under a slightly different set of conditions where energy intake is controlled, such as in inpatient studies or outpatient studies where the research team supplies all foods, energy expenditure can be calculated from energy intake and change in body energy stores. More recently there has been a growth in the number of outpatient studies where the goal is instead to estimate energy intake. In such cases, the change in body stores and energy expenditure are measured and energy intake calculated from their sum. Change in body energy stores can be measured using serial assessment of body composition. At the molecular level, fat mass (FM) accounts for the total nonessential body lipids, and the remaining components (water + protein + glycogen + mineral) together define fat‐free mass (FFM) (Wang et al. 1992). Unfortunately, change in body composition cannot always be easily measured in all studies because such measures take time, are costly, or instrumentation are not readily portable. In these cases, often only change in body weight is measured and assumptions are made about the composition of that weight change. For studies over intervals of months, especially during weight loss, there is consensus regarding the composition of that weight change (Thomas et al. 2009; Heymsfield et al. 2014). There is, however, little data available on the composition of small day‐to‐day weight variations under habitual lifestyle conditions such as using doubly labeled water as a biomarker of energy intake because knowledge is limited with respect to change in body energy stores of these short‐term fluctuations in body weight. Moreover, even when change in body composition is measured over a period as short as 2‐weeks, the individual precision of that measure limits the ability to calculate energy balance at individual level (Hall et al. 2012).

Long‐term diet restriction studies of >8‐weeks show that the change in body weight is composed of approximately three fourths FM (ΔFM/ΔWeight = ~75%) and the remaining quarter is lost as FFM (Heymsfield et al. 2014). The degree of negative energy balance causing fractional loss of FM and FFM, in these long‐term studies, are influenced by multiple factors (Heymsfield et al. 2014). Chaston et al. conducted a systematic review of published literature to identify the proportion of weight lost as FFM by various weight loss interventions. This analysis revealed that the degree of calorie restriction in <17 week studies was positively associated with percent FFM loss, with low‐calorie diets resulting in median FFM loss of 14% versus 23.4% for very low‐calorie diets. Longer term caloric restriction, however, assumed a stable level of percent FFM loss. Adding an exercise intervention to dietary restriction, as in CALERIE study led to a slightly more decrease in FM (~79%) and less FFM (21%) after 6 months as compared to diet only intervention (FM decrease by 70%; FFM decrease by 30%) (Redman et al. 2007). For studies shorter than 8‐weeks, the composition and hence energy density per kg of weight loss varies as a function of duration of the energy restriction (Heymsfield et al. 2011, 2014). For example, at 4 weeks a larger percentage of the weight lost is FFM such that the composition of weight loss is about 45% FM and 55% FFM. Adding exercise to the low‐calorie diet regimen, however, changes this ratio by increasing FM losses and reducing FFM losses after just 4 weeks of intervention (Chaston et al. 2007; Heymsfield et al. 2011). Chaston et al. (2007) reported that exercise in addition to calorie restriction achieved ~22% of fat‐free mass loss. The type and intensity of physical activity included will further impact the ΔFFM/ΔWeight ratio. Overall, existing literature shows that the 75% FM loss and 25% FFM loss is an approximation that is dependent on several variables. Because of the large differences in energy densities of FM and FFM (Wang et al. 1992), the energy density of the 4‐week changes in weight in these studies are much lower than would be estimated using the more commonly cited ratio of 75% FM and 25% FFM (Heymsfield et al. 2011; Varady 2011).

Shorter term (several days to 3 weeks) fluctuations in body weight generally range from 1 to 2 kg (Adolph 1938; Widjaja et al. 1999; Cheuvront et al. 2010; Vivanti et al. 2013). These short‐term fluctuations in body weight are assumed to be due to variations in several factors such as intestinal contents (Institute of Medicine of the National Academies, 2005; EFSA Panel on Dietetic Products, Nutrition, and Allergies [NDA], 2010), glycogen stores (Geddes et al. 1977; Fernandez‐Elias et al. 2015), labile protein stores (Swick and Benevenga 1977), and the accompanying body water (Oian et al. 1987; Ew 1996). While investigators have often reasoned that these short‐term individual daily fluctuations in body weight are primarily due to fluctuations in TBW and other components of FFM, to the best of our knowledge, there is only one published study on the composition of that variation when subjects are not making an effort to lose or gain weight (i.e., unrestricted free‐living conditions) and this study was limited to four subjects under inpatient conditions (Dole et al. 1955). Moreover, especially data under unrestricted free‐living conditions is needed to document the energy density of short‐term changes in body energy stores.

Therefore, we evaluated the variation in body composition with the variation in body weight under free‐living unrestricted conditions over a 2‐week period. Our hypothesis was that FFM as measured by DXA or calculated from the total body water would explain most of the short‐term fluctuation in body weight.

## Methods

The current analysis included data from two cohorts. First cohort (i.e., cohort 1; *n* = 24) used data from previously published work from the energy expenditure reproducibility subset of the Observing Protein and Energy Nutrition (OPEN) study and the second cohort (i.e., cohort 2; *n* = 22) used data from an ongoing study in Chicago. In both cohorts, subjects followed their habitual diets or physical activities. Changes in body weight and body composition were examined in a total of *n* = 46 adults for a period of 2‐weeks. The details of the OPEN study are described elsewhere (Subar et al. 2003; Cook et al. 2012). Briefly, the primary purpose of the study was to assess error in self‐reported dietary data using doubly labeled water (DLW) for total energy expenditure (TEE) and urinary nitrogen for protein intake. Included within the study was a subset of 24 healthy adults from metropolitan area of Washington DC in which total body water (TBW) was measured twice with dosing separated by 2‐weeks to estimate fat mass and fat‐free mass changes (Schoeller 1992). All investigations were performed in September and October 1999. The OPEN was approved by the National Cancer Institute's Special Studies Institutional Review Board and subjects provided informed consent. The ongoing study in Chicago examines the associations between sleep patterns and energy metabolism in overweight, otherwise healthy adults. The data available (between November 2015 and June 2016) under unrestricted free‐living conditions over a 2‐week period were included in the current analysis. The study was approved by the University of Chicago ethical review board and all subjects provided written informed consent. Both these studies were also approved by the University of Wisconsin Health Sciences Institutional Review board.

### Measurements

#### Body weight

Body weight was measured after an overnight fast in a hospital gown and without shoes at baseline and at the end of 2‐weeks of unrestricted free‐living period. In cohort 1, a balance beam scale was used to measure body mass during clinic visit. In cohort 2, body weight was measured using a digital weighing scale (BodyTrace, Inc., NY) with an accuracy of ±0.1 kg. It is important to note that the term “body weight” as used for cohort 1 is more of a common parlance term used to describe body mass.

#### Fat mass and fat‐free mass

In cohort 1, a dose of DLW was administered orally at ~2 g of 10 atom percent ^18^O labeled water and 0.12 g of 99.9 atom percent deuterium per 1 kg estimated total body water at baseline and the end of 2‐weeks (Subar et al. 2003; Cook et al. 2012). Three post‐dose urine specimens were collected over the next 2–4 h and analyzed by isotope ratio mass spectrometry. The isotopic dilution was used to calculate TBW (Schoeller 1992). FFM was derived from dividing the calculated TBW with a hydration constant of 0.732 (Subar et al. 2003; Cook et al. 2012). FM was calculated by subtracting derived FFM from body weight. In cohort 2, the FM and FFM were determined by dual energy X‐ray absorptiometry (DEXA) (QDR 4500 W, Hologic Inc. Arlington, MA) at baseline and at the end of 2‐weeks. All body composition measurements were performed after an overnight fast.

### Statistical analysis

All data were analyzed using SPSS (version 23.0, SPSS Inc., Chicago, IL). Results are presented as mean ± SD. Change in body weight, FM, FFM, and TBW were calculated and expressed in kg and paired t‐tests were used to test for differences between baseline and the end of 2‐week period. Pearson correlations were performed to assess the relationships between 2‐week changes in body weight with the changes in components of body composition. These correlation analyses were performed in both cohorts separately and in the combined dataset. Differences were considered significant at *P* < 0.05.

## Results

Baseline characteristics of study participants are presented in Table 1. Participants in cohort 1 were older than the participants in cohort 2. The two cohorts were similar in terms of gender distribution. Participants in both cohorts were similarly overweight.

**Table 1:** Baseline characteristics of study participants

	Cohort 1 (*n* = 24)	Cohort 2 (*n* = 22)	All participants (*n* = 46)
Age, year	55.8 ± 9.7	29.1 ± 4.9	43.0 ± 15.5
Sex (*n*, %)			
Female	11 (45.8)	9 (40.9)	20 (43.5)
Male	13 (54.2)	13 (59.1)	26 (56.5)
Height, m	1.7 **±** 0.1	1.7 ± 0.12	1.71 **±** 0.1
Baseline Weight, kg	81.3 ± 17.0	83.0 **±** 13.6	82.1 ± 15.4
Baseline BMI (kg/m^2^)	28.2 ± 4.7	27.7 ± 1.7	28.0 ± 3.6
Race/Ethnicity (*n*, %)			
White	21 (87.5)	13 (59.1)	34 (73.9)
African American	0 (0)	7 (31.8)	7 (15.2)
Other	3 (12.5)	2 (9.1)	5 (10.9)

Data are mean ± SD unless otherwise specified, BMI. body mass index.

Analysis of body weight of participants from both cohorts indicated a within subject 2‐week weight change and standard deviation of 0.18 ± 1.1 kg in cohort 1 and 0.35 ± 1.3 kg in cohort 2. In the combined dataset, the average weight change was 0.26 + 1.2 kg. In cohort 1, the average change measured in TBW was 0.15 kg. Using this TBW value, the average estimated changes in FM and FFM over 2‐week period were −0.17 kg and 0.35 kg, respectively. In cohort 2, the average changes in FM and FFM, as measured by DXA, were 0.15 kg and 0.20 kg, respectively, indicating that the subjects were on average close to energy balance. In the combined dataset, the average change in FM and FFM were −0.02 kg and 0.28 kg, respectively. Both cohorts supported the assumption that FFM hydration, that is, the ratio of TBW to FFM was stable at ~0.73 (cohort 1 FFM hydration = 0.74; cohort 2 FFM hydration = 0.75).

**Figure 1:**
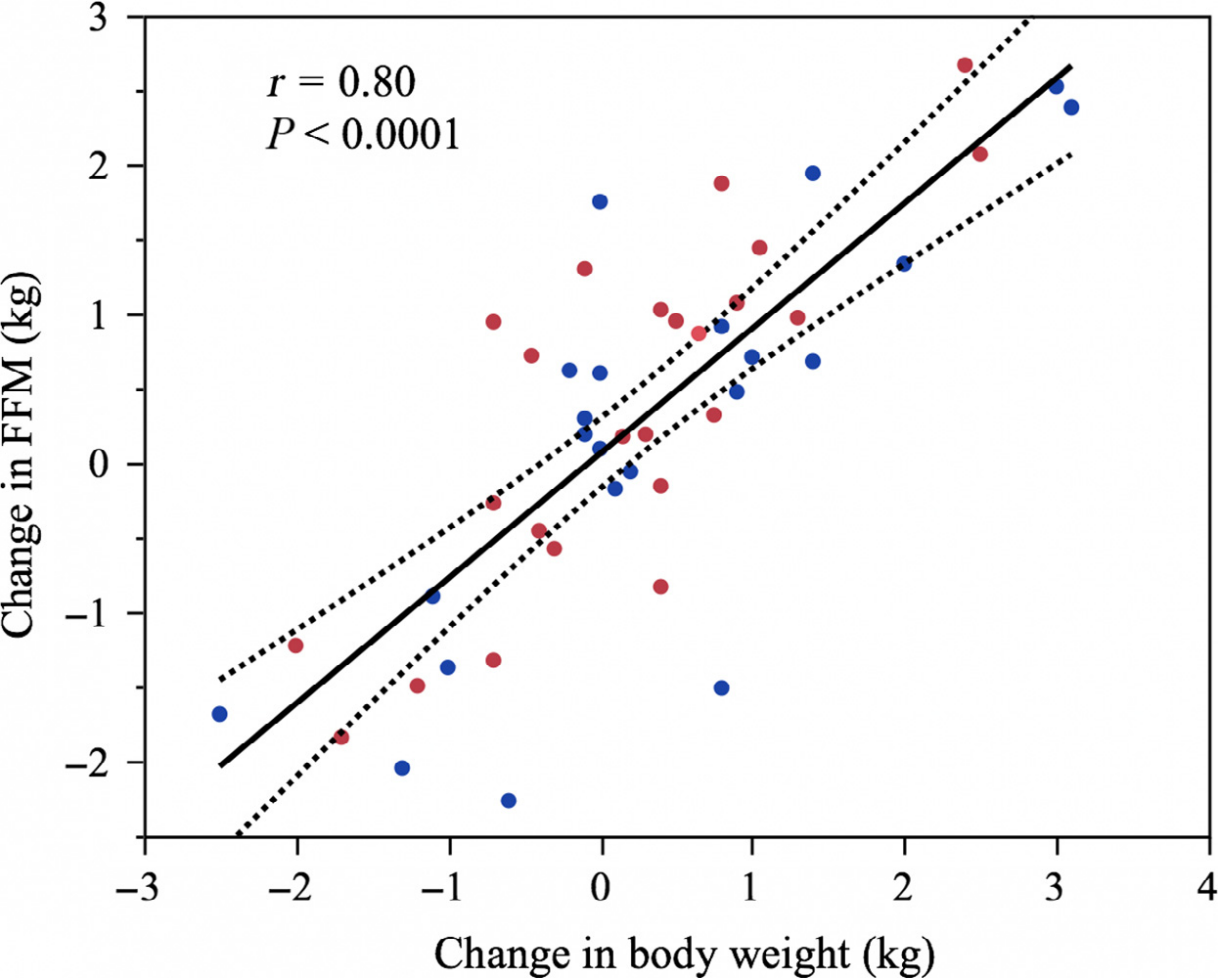
Correlation (y = 0.839x + 0.06) between 2‐week change in body weight (kg) and 2‐week change in FFM (kg) using the combined dataset from cohort 1 (*n* = 24, red dots) and cohort 2 (*n* = 22, blue dots).FFM, Fat‐free mass.

In the combined dataset, the change in body weight was significantly associated (r = 0.80, *P* < 0.0001) with the change in FFM (Fig. 1). The slope indicated that every 1 kg change in body weight was composed of 0.84 kg or 84% FFM. To calculate the FM composition per kg of body weight change, we subtracted the FFM estimate (0.84 kg) obtaining FM estimate of 0.16 kg. Using tissue energy densities of 1020 kcal/kg and 9500 kcal/kg for FFM and FM, respectively (Thomas et al. 2010), we multiplied the estimates for FM and FFM with their respective energy densities and sum them up to obtain the energy density of total body weight. This calculation indicated that in the combined dataset, the average energy densities of 2‐week changes in body weight was 2380 kcal/kg.

Additionally, in the combined dataset, examining the correlations between 2‐week changes in body weight and FM or FFM in men and women indicated that 84% of change in body weight was FFM in men, versus 78% of the change in body weight was FFM in women. However, this small difference in the composition of the changes in body weight between genders did not reach statistical significance (*P* = 0.196).

## Discussion

We demonstrated that short‐term variations in body weight in subjects who are not making an effort to lose or gain weight (i.e., under unrestricted free‐living conditions) were largely composed of changes in FFM and its major compartment of water. The change in energy density over 2‐week period was 2.4 kcal/g in our study in the combined dataset. Dole et al. (1955) reported a similar change in energy density of 2.5 kcal/g in four obese women after 14 days of inpatient paradigm with carefully controlled energy intake and physical activity . As expected, the relative contribution from FFM is much larger than those summarized for systematic changes in weight in a 12‐week or longer period, which indicates a much lower energy density than those reported for systematic over‐ or under‐feeding (Casper et al. 1990; Heymsfield et al. 2014).

Although there was a strong association between change in FFM or TBW and change in body weight, we found no association of change in FM with body weight change (data not shown). This is not surprising, however, because in both cohorts the average FM changes were small and thus not significant due to limited power for detecting small changes in FM. Our slightly lower values for change in FM and slightly larger values for change in FFM as compared to what has been reported in medium‐term energy restriction however, can be explained by the absence of a prescribed energy restriction in our study as compared to research where subjects were systematically in negative energy balance (Heymsfield et al. 2014; Muller et al. 2015).

Our results also indicate that FFM is primarily responsible for the majority of daily fluctuations in body mass during near energy balance conditions. Any variation in components of FFM, such as muscle, organ, bone or total body water may impact change in FFM and therefore body weight. To study the effect of 3‐week calorie restriction on adaptive thermogenesis and body composition, Müller and colleagues duplicated the early portion of the Minnesota starvation experiment in normal weight adults (Muller et al. 2015). These investigators performed a full body MRI to study the change in organ mass with calorie restriction and reported that the loss of FFM was explained by a decrease in skeletal and liver/kidney mass (Muller et al. 2015). In particular, each gram of protein in the organ mass was accompanied by 1.6–3.35 g of water and therefore contributed to daily changes in FFM (Heymsfield et al. 2011). It is important to note that FFM is composed of approximately 70–75% of total body water, that is, the hydration factor (Wang et al. 1999). It is estimated that in healthy individuals the total body water can fluctuate up to ±5% daily (Ew 1996). Therefore, any gain or loss in the total body water has a large impact on the daily variations in FFM and therefore body weight.

Body stores of glycogen in muscle, liver, and fat cells are hydrated with three to four parts water. Geddes and colleagues in their early studies analyzed liver glycogen molecule size in vitro and showed that the each gram of glycogen had 1.5–2.7 g of water attached to it (Heymsfield et al. 2011). Similarly, each gram of glycogen is stored in human muscle with 3 grams of water (Fernandez‐Elias et al. 2015). The high skeletal muscle glycogen levels increase the water content of FFM primarily due to osmotic pressure exerted by glycogen granules within the muscle sarcoplasm. Investigators have shown that in study participants following a very low‐calorie diet, glycogen mobilization also mobilized the water associated with the stores leading to rapid weight loss (Kreitzman et al. 1992). Daily variation in carbohydrate intake or utilization in both our datasets and the associated water may have been a primary contributor to the observed FFM fluctuation.

Changes in intestinal contents would also be expected to contribute to short‐term weight fluctuation. Gut contents and fecal water contribute to a variability in 100‐300 ml/day water in the fasted state (Institute of Medicine of the National Academies, 2005; EFSA Panel on Dietetic Products, Nutrition, and Allergies [NDA], 2010). These ranges, however, may vary with change in ambient temperature and physical activity level (Institute of Medicine of the National Academies, 2005; EFSA Panel on Dietetic Products, Nutrition, and Allergies [NDA], 2010). Physical activity may also impact FFM hydration factor in the body at warm ambient temperatures. In a study by Baker et al. (2009), eight endurance athletes completed 2 h of interval training in hot temperature followed by a run to exhaustion, to test the change in total body water using deuterium oxide dilution method. Investigators reported a significant correlation between change in body mass and TBW, showing that much of the change in body mass with physical activity is contributed by the TBW.

There are limitations to our findings. The body composition analysis was based on the two‐compartment model. The close agreement between the two cohorts using two different body composition techniques, namely TBW and DXA, however, indicates that these two‐compartment hydration assumptions were not violated. Furthermore, because the studies in both cohorts were performed under free‐living conditions, we did not control the external variables such as, variation in diet, external temperature or physical activity, or menstrual changes for women, and thus could determine specific causes for individual variations in FFM that we observed. Although, our sample size was small for a formal assessment of gender differences, the analyses of change in body weight separately in men and women, did not indicate a significant influence of gender on body composition change in our combined dataset.

Although our combined dataset included some subjects who gained weight while losing FFM and some other who lost weight while gaining FFM these results are not surprising for these small changes in weight given the near precision limits of body composition methods. For example, while isotope dilution is a standard method for body composition measurement, to attaining a precision of <1% in the measurement of FFM from TBW requires close attention to technical details. Any slight imprecision in any aspect of measurement including dose preparation, administration of tracer dose, sample collection, isotope analysis, and several other experiment specific variables may lead to random errors of hundreds of grams (Bhutani et al. 2015). The precision of DXA is also on the order of hundreds of grams for FFM and FM and this becomes a limiting factor because body composition measures are not as precise as the measurement of body weight. Muller et al. (2012) showed that all body composition methods are limited by random error of measurement, that is, the imprecision and errors involved in practical applications that limit the minimal detectable change in outcome variable . One should not expect that weight change alone in free‐living individuals can distinguish between total energy balance and long‐term negative or positive energy balance of less than 500 kcal/d in an individual. This is primarily because in both of these conditions, body weight will not change by more than 1 or 2 kg over a 2‐week period. Based on our findings, in subjects who report not consciously attempting to lose or gain weight, short term changes in body weight would be expected to largely composed of FFM. Thus, for subjects under unrestricted, free‐living conditions (i.e., without systematic long‐term weight change), the energy density for small short‐term fluctuations in body weight would correspond to about 2380 kcal/kg or about 2 kcal/g. As such the contribution of energy stores to the energy balance equation appears to be small (Thomas et al. 2009; Hall et al. 2011).

In summary, we report a relatively larger contribution of FFM (84%; mainly body water), as compared to FM (16%), to fluctuations in body weight over 2‐weeks under unrestricted free‐living conditions. We also show that the energy density of short‐term changes in body energy stores is small. Our findings provide important insights to short‐term, near energy balance studies where body composition cannot be easily measured.

## Conflict of Interest

All authors declare no conflict of interests
